# Daily Symptom Home Monitoring Decreases Hospital Readmissions in Children and Young Adults With Acute Lymphoblastic Leukemia

**DOI:** 10.1002/cam4.71719

**Published:** 2026-04-08

**Authors:** Rotem Fishel Ben‐Kenan, Laura L. Retson, Briana Fodor, M'hamed Temkit, Alexandra Walsh

**Affiliations:** ^1^ Center for Cancer and Blood Disorders, Phoenix Children's Hospital Phoenix Arizona USA; ^2^ Department of Child Health University of Arizona College of Medicine Phoenix Arizona USA; ^3^ Department of Clinical Research, Phoenix Children's Hospital Phoenix Arizona USA

**Keywords:** acute lymphoblastic leukemia, readmission rates, symptom monitoring, technology intervention

## Abstract

**Objective:**

Advancements in treatment of Acute Lymphoblastic Leukemia have led to high overall survival rates but are accompanied by significant treatment‐related morbidity and mortality. We aimed to determine whether daily symptom home monitoring paired with targeted nursing intervention decreased readmission rates in newly diagnosed pediatric and adolescent young adults with Acute Lymphoblastic Leukemia at a single large pediatric oncology center.

**Methods:**

A daily symptom home monitoring survey was texted to families' cellular phones of patients undergoing the initial chemotherapy block (Induction) for Acute Lymphoblastic Leukemia, and responses were monitored and intervened on by nurse clinicians. Control families did not receive daily symptom home monitoring surveys. Readmission rates, total days at home, patient symptoms, and quality of life measures were assessed in both control and participant groups.

**Results:**

Eighteen control patients and 58 participants were enrolled. Among the patients in the intervention group, 48 patients (82.8%) were not readmitted vs. 10 patients (55.6%) in the control group (*p* = 0.028). Patients in the intervention group spent 18.7 days at home versus patients in the control group spent 16.4 days at home (*p* = 0.112). Patient demographics and caregiver distress measures did not significantly differ between the two groups.

**Conclusions:**

Implementation of a daily symptom home monitoring texted survey aimed at identifying and responding to patients' morbidities allowed for timely intervention and significantly decreased readmission rates without worsening caregiver distress.

**Abbreviations**
ALL: Acute lymphoblastic leukemia
AYA: Adolescent and young adult
HIPAA: Health insurance portability and accountability act of 1996
HRQL: Health related quality of life
IRB: Institutional review board
LARs: Legally authorized representatives
NCCN: National comprehensive cancer network

## 1 Introduction

Improvements in treatment and supportive care have increased overall childhood cancer cure rates to about 85%. Acute lymphoblastic leukemia (ALL) is the most common childhood cancer, representing 75%–80% of acute leukemias in pediatric patients [1, 2]. Overall survival rates are approaching 90% for acute leukemias with improvements in risk‐stratification and addition of targeted therapies such as blinatumomab for B cell ALL [3, 4]. However, these high cure rates are accompanied by significant treatment‐related morbidity and mortality. The first block of chemotherapy for ALL (“Induction”) is an especially high risk phase of treatment which consists of 28 days of intensive therapy designed to markedly reduce the leukemia burden by killing as many cancer cells as possible [1]. Unfortunately, patients suffer significant morbidity during induction therapy with high rates of infection, chemotherapy indued nausea/vomiting, fatigue, pain, deconditioning, coagulopathy, nervous system complications such as neuropathy, gastrointestinal complications such as constipation and pancreatitis, and side effects attributable to steroids including but not limited to weight gain, gastritis, hypertension, and hyperglycemia [5, 6, 7, 8, 9]. Many of these treatment‐related complications lead to readmissions during induction therapy which are both burdensome for newly‐diagnosed patients and their families as well as costly for the health care system.

Previous studies have highlighted the impact on quality of life and burden for newly diagnosed patients and their families. In a recent study by Parker et al. in 2025 assessing 442 parents perspective on the difficulties associated with their child's leukemia treatment, it was reported that 353/442 (79.9%) of parents found unplanned hospital/ER visits one of the most difficult and disruptive components of therapy, with 333/442 (75.3%) of parents reporting the same for unplanned hospital overnight stays [10]. Another study in 2020 by Huang et al. highlighted how increased symptom burden is associated with poorer child health‐related quality of life and in turn associated with more family strain [11]. Overall, low quality of life among caregivers of childhood leukemia patients has been well documented in the literature with effects on social relationships, physical health, psychological well‐being and financial security [12, 13, 14]. While there is a paucity of data on the specific cost burden related to hospital readmissions for patients with ALL undergoing therapy, Wedekind et al. in 2016 showed that the median length of stay for ALL induction admissions varied from 5 to 31 days across hospitals and the thirty‐day median inpatient costs per patient ranged from $32,000 for short length of stay (< 7 days), $40,000 for medium length of stay (8 to 15 days) and $47,000 for long length of stay (> 16 days) [15].

In a previous analysis of newly diagnosed ALL patients at Phoenix Children's Hospital between 2018 and 2020, 48 patients out of 98 (49%) were readmitted during Induction for a total of 57 hospital readmissions. Of these readmissions, 27 (47.4%) were due to fever and deemed not preventable. However, 16 readmissions (28.1%) were deemed preventable as they were secondary to gastrointestinal complaints such as constipation, pain, and deconditioning. An additional nine readmissions (15.8%) were deemed possibly preventable (as adjudicated by the study team) due to hyperglycemia, hypertension, nephrolithiasis, and weight loss/poor oral intake [16].

Significant work has been done in collecting symptom data in pediatric cancer patients. In 2024, Dupuis et al. Published the results of a randomized cluster trial involving 20 pediatric cancer centers in the US. This study implemented a thrice‐weekly symptoms screening with symptom feedback and management of care pathways. Patients enrolled at the centers randomized to the intensive symptom screening protocols experienced improved symptom scores and increased symptom‐specific interventions, thereby suggesting that symptom screening should be integrated into routine clinical care [17]. The importance of symptom screening and its potential clinical benefits have also been illustrated in adult cancer patients where studies have shown that symptom screening leads to improved symptom control, supportive care measures, and patient satisfaction [18] as well as fewer ER visits and hospitalizations, enabling patients to remain on chemotherapy longer, and even prolonging survival [19]. Basch et al. evaluated adults with advanced cancer who were randomized to receive web‐based symptom reporting with automated clinician e‐mail alerts versus standard‐of‐care symptom management. The intervention group had better Health Related Quality of Life (HRQL), fewer ER visits, and fewer hospitalizations, and improved overall survival of 31.2 months compared to the control group of 26 months (*p* = 0.03) [20].

In this study, we sought to investigate the clinical benefit of implementing daily home monitoring via a secure texting interface for symptom screening during induction for newly diagnosed pediatric patients with ALL. Our primary endpoint was the rate of readmissions during Induction therapy. We also hypothesized that daily interactions between families and the study team might decrease caregiver distress as measured by the National Comprehensive Cancer Network (NCCN) distress thermometer [21]. In addition, by collecting reported symptoms data daily, we hoped to better understand daily symptom burden in patients with newly diagnosed pediatric ALL, which could then inform future interventions.

## 2 Methods

### 2.1 Study Design

This was a single institution prospective cohort study conducted at Phoenix Children's Hospital and approved by the Institutional Review Board (IRB). We worked closely with our institution's information technology department on the creation and development of a daily symptom home monitoring system utilizing a secure, HIPAA‐compliant texting interface platform in which symptom questionnaires would be sent daily to parents/legally authorized representatives (LARs) or patients themselves if > 18 years old (LARs—hereafter noted as “family”) of newly diagnosed patients with acute lymphoblastic leukemia during induction therapy. The specific questions chosen for the daily symptom survey were specifically aimed at addressing the preventable and possibly preventable reasons for re‐admission that were identified in our previous analysis of newly diagnosed ALL patients undergoing induction therapy [16]. These preventable and possibly preventable reasons for re‐admission—and the focus of our survey questions—included nausea, vomiting, decreased oral intake, constipation, pain, and compliance with chemotherapy [10]. We did include one question regarding fever despite fever being a non‐preventable cause for re‐admission as we felt this is important to monitor for given our high‐risk patient population. In addition, the survey interface provided a free text box for families to express specific concerns, thereby allowing for direct communication between families and the medical team to review concerns not specifically addressed via the survey questions.

The study was subsequently implemented in two phases. Phase one consisted of recruitment of families for the control group cohort between June and November of 2022. The control group received a weekly questionnaire via the texting interface regarding caregiver distress and quality of life measures on days 8, 15, 22, and 29 of treatment (full text of administered surveys is available in Supplement 1). The control group did not receive the daily symptom questionnaire. During Phase 2 (from November 2022 to September 2024), patients were recruited and consented to the intervention portion of the trial. The intervention group was sent daily questionnaires via the texting interface to assess patient symptoms, as well as the same weekly questionnaires measuring caregiver distress and quality of life measures as administered to the control group. Per institutional policy [10], all patients were inpatient for the initial 7 days after starting chemotherapy. Therefore, families received a maximum of 21 daily surveys in addition to the 4 weekly surveys. All guardians provided informed consent, and patients provided assent, when possible, given the patient's medical condition and cognitive level.

### 2.2 Study Population

Potential patients were identified and eligibility assessed at weekly meetings with all leukemia providers. English or Spanish speaking patients with newly diagnosed Acute Lymphoblastic Leukemia or Lymphoma, who had not yet completed their first week of therapy and who had a working cellular telephone able to receive text messages were eligible for inclusion. Patients who had relapsed disease, were previously treated with any chemotherapy, were neither English nor Spanish speaking, or planning to transfer care out of the institution after initial hospitalization were excluded. In addition, patients with infantile ALL, myeloid, mixed phenotype, or ambiguous lineage leukemias were excluded (Table 1).

**TABLE 1:** Inclusion and exclusion criteria.

Inclusion criteria	Exclusion criteria
Patients newly diagnosed with ALL who have not yet begun their first month of induction therapy	Patients with relapsed disease
English or Spanish speaking	Previously treated with chemotherapy
	Non‐English or Spanish speaking
	Myeloid, Mixed phenotype, or ambiguous lineage leukemias
	Infantile leukemia
	Plan to receive care outside of PCH

### 2.3 Study Procedures and Data Collection

Eligible families were recruited and consented to participate by research staff during their initial admission following diagnosis for ALL Participants and/or guardians provided their primary phone number to receive daily texts. Demographic, disease, and treatment variables were collected from the hospital's electronic medical record and included gender, race, ethnicity, preferred language, age at diagnosis, subtype of acute lymphoblastic leukemia or lymphoma (B‐cell vs. T‐cell), treatment protocol, and CNS status. Any patient admitted throughout induction was removed from the study cohort.

Readmission data (date of admission and discharge, number of days admitted, ICU admission, reason for admission and death if applicable) were recorded from the electronic health record. Data were stored in Redcap, a secure online platform for building and managing online databases through the University of Arizona. For any deaths, information regarding timing and cause of death was recorded.

All surveys were translated by the Phoenix Children's Hospital Language Services department, and family members could toggle between Spanish and English language surveys as applicable.

**FIGURE 1:**
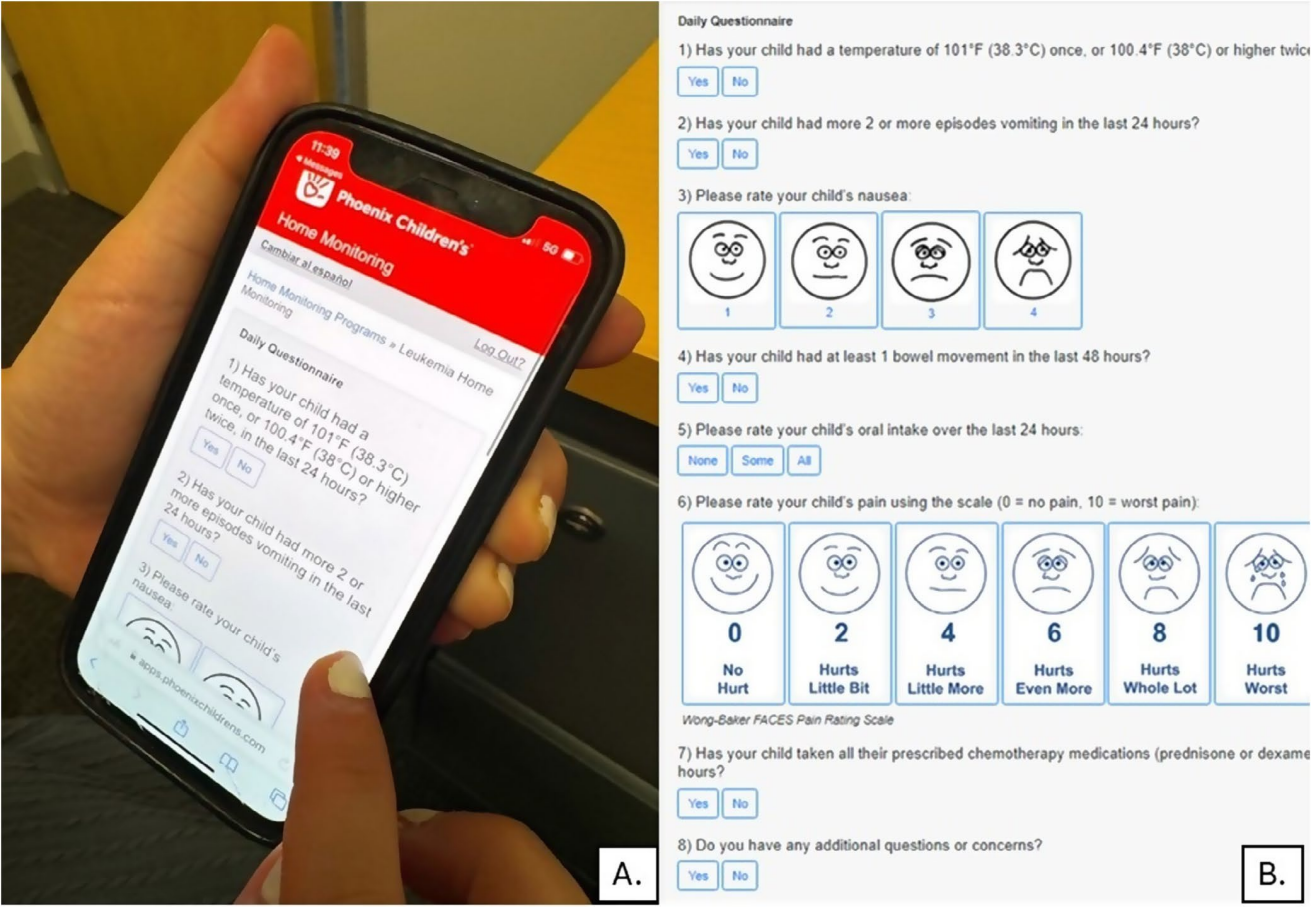
Participant and/or Guardian View of Daily Questionnaire via Secure Texting Interface. Participant and/or guardian view (A) of the home monitoring system via secure texting interface. Full daily questionnaire that is sent on day 8 through day 29 of induction therapy (B). Note: The Pediatric Nausea Assessment Tool (PeNAT) [22] was utilized to assess nausea and the Wong‐Baker FACES Pain Rating Scale to measure pain.

The control cohort received a weekly questionnaire regarding caregiver distress and quality of life measures on day 8, 15, 22, and 29 of treatment. In the intervention group, consenting participants also received the weekly questionnaire regarding caregiver distress and quality of life measures on day 8, 15, 22, and 29 of treatment. In addition, they were enrolled in a secure and HIPPA‐compliant texting interface platform to receive daily questionnaires via text message to their smartphone regarding symptoms that began on day 8 of treatment and continued through day 29 (Figure 1). The Pediatric Nausea Assessment Tool (PeNAT) [23] was utilized to assess nausea and the Wong‐Baker FACES Pain Rating Scale to measure pain. The survey also included a free‐text box to allow families to communicate directly with the medical team. Texts were sent at 9 am each morning, 7 days a week. Completion of the questionnaire triggered an email sent to the research team and leukemia nurse clinicians.

**FIGURE 2:**
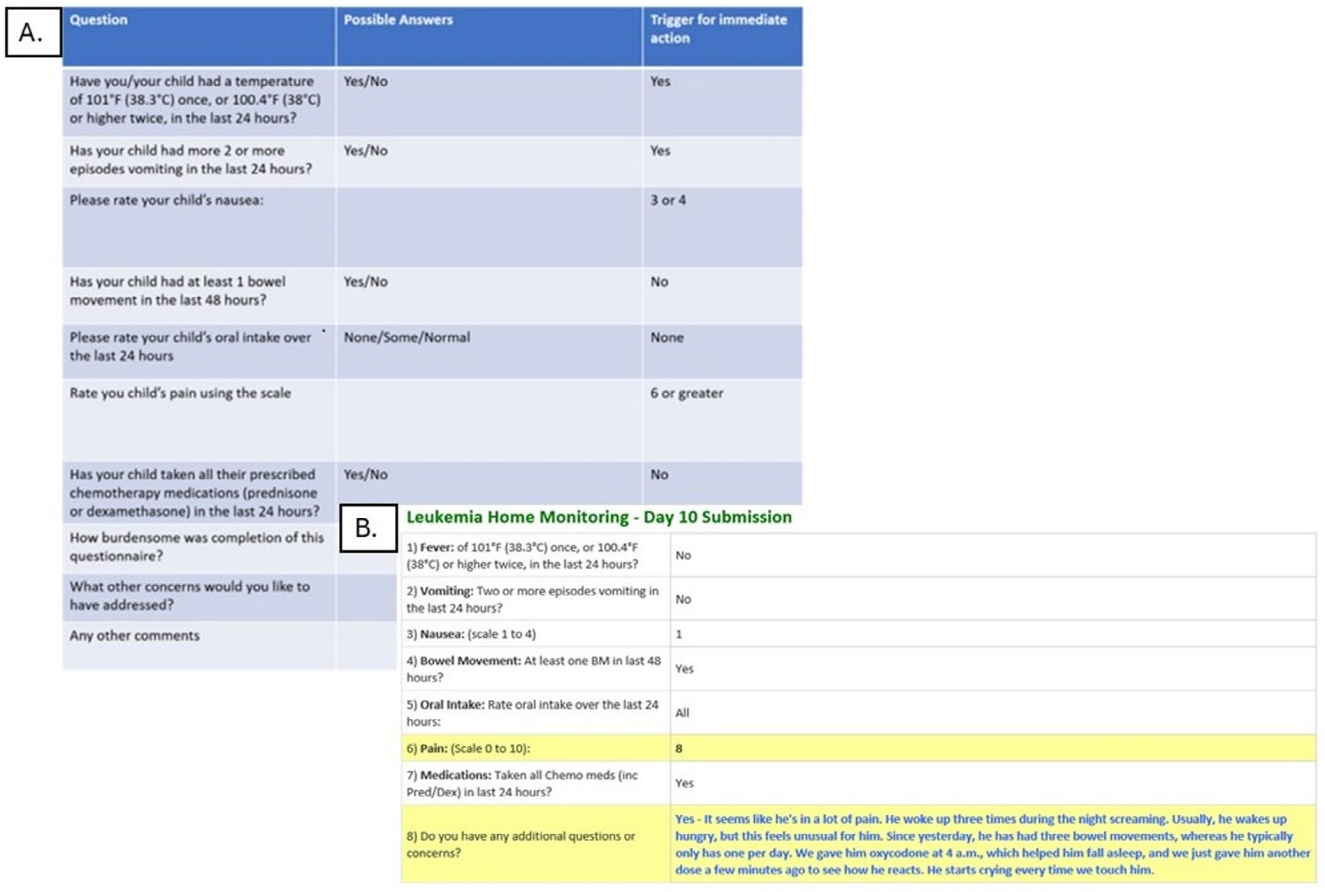
Provider View of Daily Symptom Home Monitoring System. Daily questionnaire answers that trigger immediate action by leukemia provider (A) including calling participant and/or guardian to discuss symptoms of concern and allow for early intervention. Provider view displaying highlighted concerning daily questionnaire answers that trigger for immediate action (B).

Threshold values of concern were determined by the study team a priori and resulted in those rows in the email to the study team to be highlighted, as shown in (Figure 2). If the family indicated the presence of a fever, they would immediately receive an automated message to take the patient to the nearest emergency department rather than waiting for nursing feedback. A member of the study would contact the family member in real time to discuss the patient's symptom(s) of concern and allow for early and timely intervention. One call was made per patient for any number of triggering responses in the questionnaire they returned. During regular clinic hours and on the weekends, between 8 am and 5 pm, a member of the assigned dedicated nursing team monitored their inbox at least once an hour. During evenings, a member of the assigned dedicated nursing team monitored their inbox less frequently and called the following morning. However, extensive education with the teach‐back method was incorporated during the enrollment process to ensure patients and their families understood that if there were any urgent concerns to call the on‐call oncologist. Lastly, we assessed whether this was burdensome to the medical team by meeting on a weekly basis for verbal feedback.

### 2.4 Statistical Analysis

Data were summarized using frequencies and percentages for categorical variables and means, standard deviations, and ranges for continuous variables. Between‐group comparisons were conducted using the chi‐square test or Fisher's exact test, as appropriate, for categorical variables, and the Wilcoxon rank‐sum test for continuous outcomes. Within‐subject analyses were performed using a two‐factor repeated‐measures model with day (8, 15, 22, and 29) and group (intervention vs. control) as fixed effects, including the day × group interaction. Generalized estimating equations (GEE) were used to account for within‐subject correlation cross repeated measurements. All tests were two‐sided with a significance level of α = 0.05. Statistical analyses were performed using SAS version 9.4 (SAS Institute, Cary, NC).

## 3 Results

**FIGURE 3:**
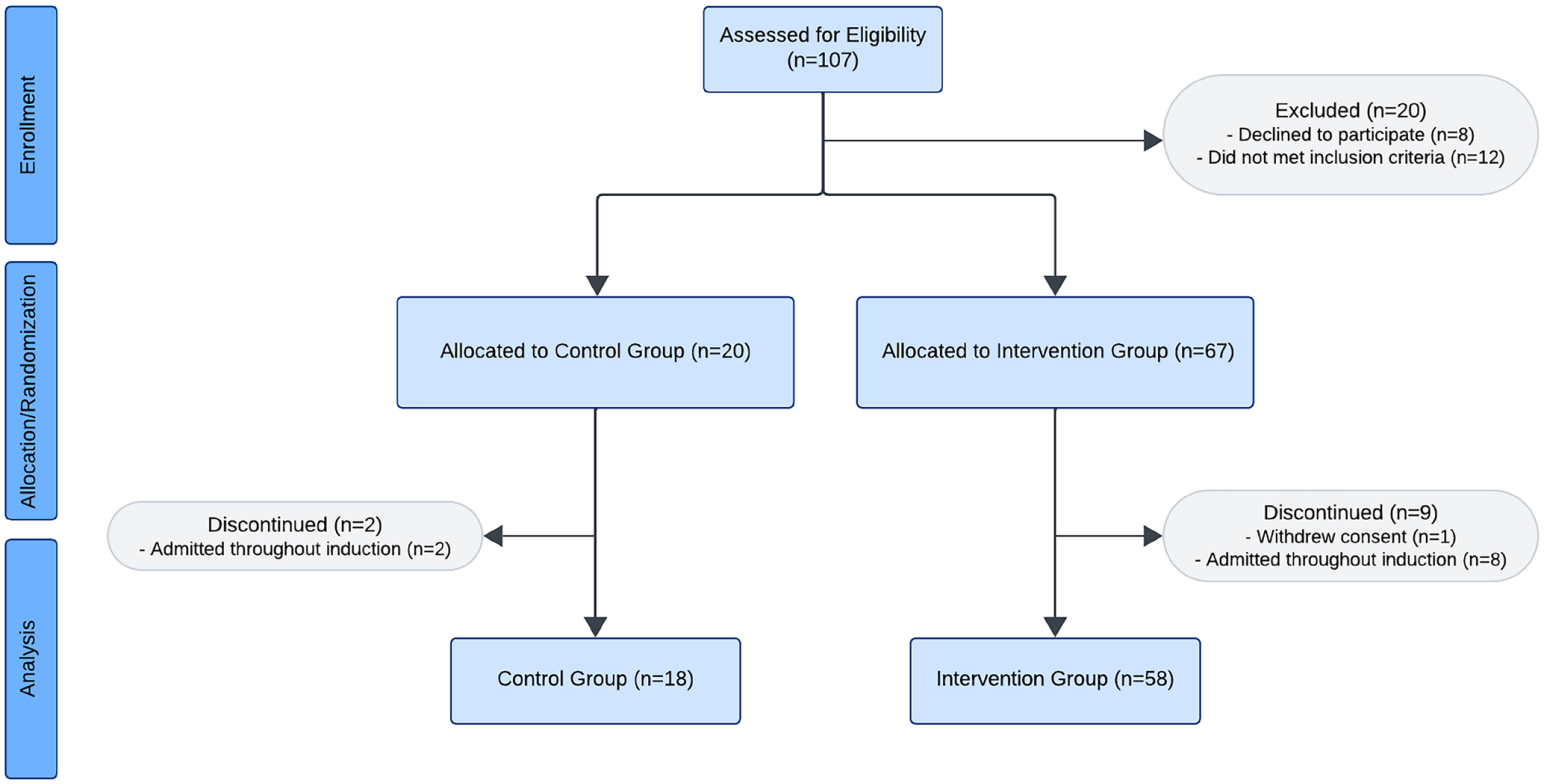
Consort Diagram. A total of 107 patients were assessed for eligibility between June 2022 and September 2024. Twenty patients were excluded during the enrollment period as they had not met inclusion criteria (*n* = 12) or declined to participate (*n* = 8). Subsequently, 20 patients were allocated to the control group and 67 patients to the intervention group. In the control group, two patients were discontinued as they remained admitted throughout induction, thereby resulting in analysis of 18 patients in the control group. In the intervention group, 9 patients were discontinued due to consent withdrawal (*n* = 1) or remaining admitted throughout (*n* = 8), thereby resulting in analysis of 58 patients in the intervention group.

In total, 107 patients were assessed for eligibility between June 2022 and September 2024. Twenty patients were excluded during the enrollment period as they did not meet inclusion criteria (*n* = 12) or declined to participate (*n* = 8). The first 20 patients diagnosed with ALL were allocated to the control group, and the subsequent 67 patients to the intervention group. Two patients were removed from the control group as they remained admitted throughout induction, leading to the analysis of 18 patients. In the intervention group, 9 patients were removed from the study due to consent withdrawal (*n* = 1) or admission throughout induction (*n* = 8), resulting in analysis of 58 patients (Figure 3).

**FIGURE 4:**
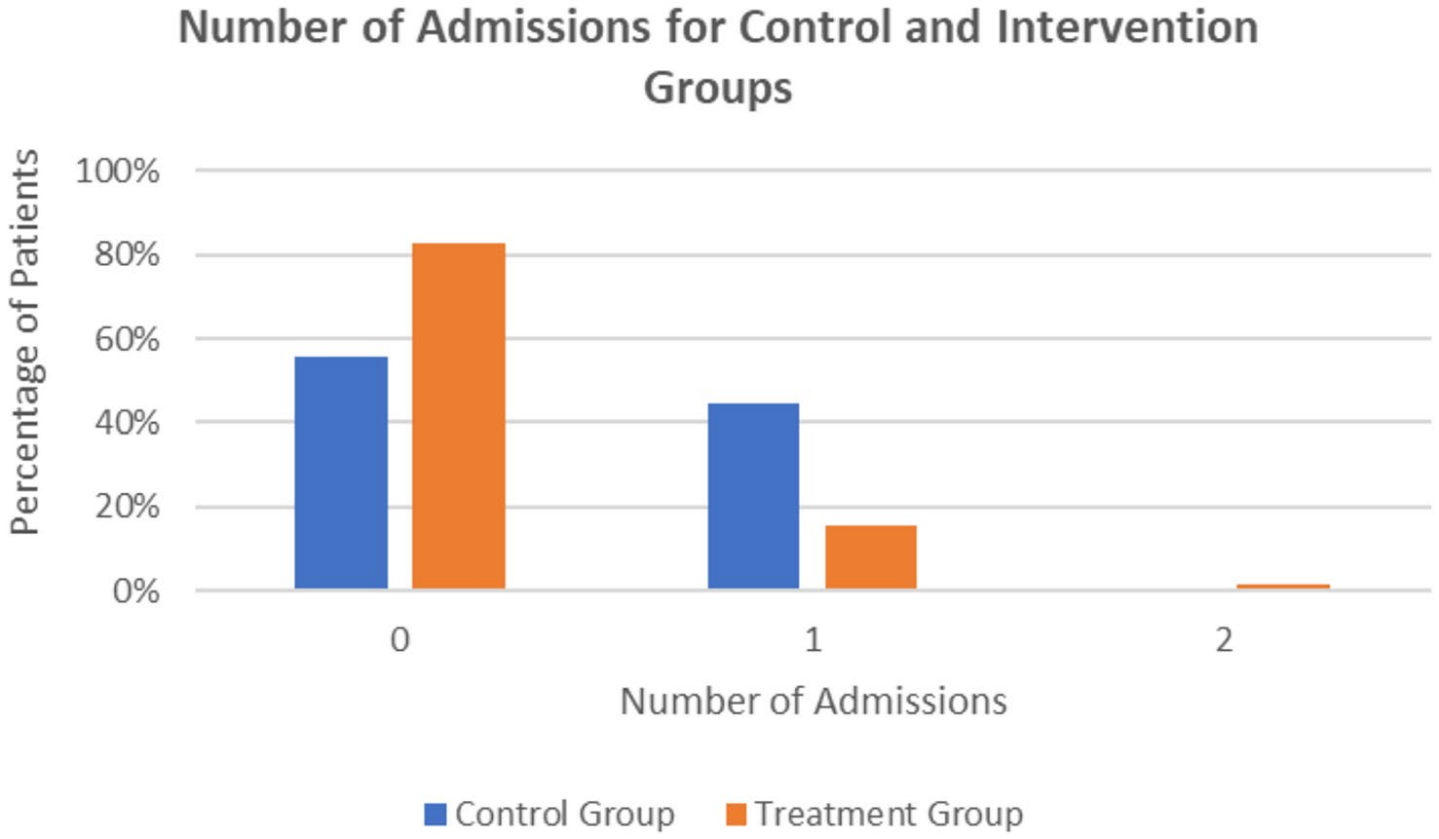
Number of Admissions for Control and Intervention Groups. In the control group, 10 patients (55.6%) had zero admissions, 8 patients (44.4%) had one admission, and no patients (0%) had two admissions. In comparison, the intervention group had a greater percentage of patients (*n* = 48, 82.8%) with zero admissions, a smaller percentage of patients (*n* = 9, 15.5%) with one admission, and one patient (1.7%) with two admissions.

Patient demographics are shown in Table 2. The control group consisted of 12 male (66.7%) and 6 female (33.3%), and the intervention group consisted of 33 male (56.9%) and 25 female (43.1%). There was no significant difference in mean age at diagnosis between the groups, 8.43 ± 5.61 years for the control group and 8.49 ± 6.14 years for the intervention group. Most patients in both groups were Caucasian (12 patients (66.7%) in control group, 51 patients (87.9%) in intervention group) and nine patients (50%) identified as Hispanic ethnicity in the control group while 19 patients (32.8%) identified as Hispanic ethnicity in the intervention group. Overall, most patients in this study were diagnosed with B‐cell ALL as compared to T‐cell ALL (15 patients (83.3%) with B‐cell ALL in control group, 51 patients (87.9%) with B‐cell ALL in intervention group) and most patients were CNS 1 status (13 patients (72.2%) in control group, 40 patients (68.9%) in intervention group). In the control group, seven patients (38.9%) were treated with AALL 1731 (standard‐risk‐3‐drug induction) as compared to 20 patients (50%) in the intervention group. Eight patients (44.4%) of patients in the control group were treated with AALL 1732 (high‐risk 4‐drug induction) compared to 23 patients (39.7%) in the intervention group. Three patients (16.7%) in the control group were treated with AALL 1231 (also utilizing 4‐drug induction) compared to 6 patients (10.3%) in the intervention group. Of note, this highlights that there was a relatively large difference in proportion of patients with SR B‐ALL between the control and intervention groups compared to the HR B‐ALL and T‐ALL patient populations who were more evenly distributed between the control and intervention cohorts. In total, there were seven patients (38.9%) in the control group who received a 3‐drug induction compared to 29 patients (50%) in the intervention group, and a total of 11 patients (61.1%) who received a 4‐drug induction (treated with AALL 1732 or AALL 1231) compared to 29 patients (50%) in the intervention group. No statistically significant differences were noted between the two groups for any variable (Table 2).

**TABLE 2:** Patient Demographics.

	Control group (*N* = 18)	Intervention group (*N* = 58)	*p*
Sex			0.586^a^
Male	12 (66.7%)	33 (56.9%)	
Female	6 (33.3%)	25 (43.1%)	
Race			0.410^a^
Caucasian	12 (66.7%)	51 (87.9%)	
African American	0 (0%)	1 (1.7%)	
Asian	1 (5.6%)	2 (3.4%)	
Native American	2 (11.1%)	2 (3.4%)	
Other/Unknown	3 (16.7%)	2 (3.4%)	
Ethnicity			0.263^a^
Hispanic	9 (50.0%)	19 (32.8%)	
Non‐Hispanic	9 (50.0%)	39 (67.2%)	
Preferred Language			0.497^a^
English	16 (88.9%)	46 (79.3%)	
Spanish	2 (11.1%)	12 (20.7%)	
Type of ALL			0.693^a^
B cell	15 (83.3%)	51 (87.9%)	
T cell	3 (16.7%)	7 (12.1%)	
Treatment Protocol			0.557^a^
AALL1731	7 (38.9%)	29 (50.0%)	
AALL1732	8 (44.4%)	23 (39.7%)	
AALL1231	3 (16.7%)	6 (10.3%)	
# of drugs in induction			0.434^a^
3‐drug induction (AALL 1731)	7 (38.9%)	29 (50.0%)	
4‐drug induction (AALL 1732 or AALL 1231)	11 (61.1%)	29 (50.0%)	
CNS status*			1.000^a^
Unknown	0 (0%)	1 (1.7%)	
1	13 (72.2%)	40 (68.9%)	
2	4 (22.2%)	15 (25.9%)	
3	1 (5.6%)	2 (3.4%)	
Age at diagnosis			0.956^b^
Mean (SD)	8.43 (5.61)	8.49 (6.14)	
Range	(1.14–17.45)	(1.37–25.56)	

^a^ Fisher's exact.

^b^ Wicoxon rank‐sum.

* Per COG protocols, CNS 1: In CSF, absence of blasts on cytospin preparation, regardless of the number of WBCs. CNS 2: In CSF, presence of < 5/uL WBCs and cytospin positive for blasts, or traumatic LP, or > 5/uL WBCs, cytospin positive for blasts, but negative by Steinherz/Bleyer algorithm. CNS 3: In CSF, presence > 5/uL WBCs and cytospin positive for blasts and/or clinical signs of CNS leukemia.

Data collected from the daily symptom questionnaires is reported in Table 3. Most patients reported no vomiting (98.3%), a mean nausea level of 1.24 ± 0.52 (on a scale of 1–4), regular bowel movements (96.4%), good oral intake (88.2%), a mean pain level of 1.46 ± 1.73 (on a scale of 1–10), and excellent compliance with oral home medications as prescribed (98.9%). There were 512 discrete responses triggering immediate action and 342 calls were made by the study team.

**TABLE 3:** Patient Symptoms.

	Intervention group (*N* = 788 symptom reports)
Vomiting	
No	772 (98.3%)
Yes	13 (1.7%)
Nausea (scale 1–4)	
Mean (SD)	1.24 (0.52)
Range	1–4
Bowel movements	
No	28 (3.6%)
Yes	757 (96.4%)
Oral intake	
all	692 (88.2%)
some	86 (11.0%)
none	7 (0.9%)
Pain	
Mean (SD)	1.46 (1.73)
Range	(0–8)
Taking all medications	
No	9 (1.1%)
Yes	775 (98.9%)

Patient outcomes are shown in (Table 4, 5). In the control group, 8 patients (44.4%) required one re‐admission during the study period as compared to 9 patients (15.5%) in the intervention group (*p* = 0.028) (Figure 4). Although patients enrolled in the intervention group spent more days at home, the difference was not statistically significant the control group had a mean of 16.39 days at home out of a possible 21 days, versus 18.74 days for the treatment group (*p* = 0.112). Analysis of patient outcomes by comparing patients who received standard‐risk 3‐drug induction (undergoing treatment with AALL 1731) and patients who received high‐risk 4‐drug induction (undergoing treatment with AALL 1732 or AALL 1231) is shown in Table S2. In the cohort patients receiving 4‐drug induction, there was a statistically significant higher rate of re‐admission compared to patients receiving a 3‐drug induction (mean 0.35 versus 0.14 respectively, (*p* = 0.0083)). Specifically, 14 patients (35%) in the 4‐drug induction cohort required one readmission during the study period as compared to 3 patients (8.3%) in the 3‐drug induction cohort. Overall, 88.9% of patients receiving a 3‐drug induction regimen had no readmissions while only 65% of patients receiving 4‐drug induction required no re‐admissions. This is overall consistent with the literature that patients receiving intensified regimens are at higher risk of hospitalizations [22].

**TABLE 4:** Patient Outcomes.

	Control group (*N* = 18)	Intervention group (*N* = 58)	*p*
Number of admissions per patient			**0.028** ^a^
0	10 (55.6%)	48 (82.8%)	
1	8 (44.4%)	9 (15.5%)	
2	0 (0%)	1 (1.7%)	
Total admission number			**0.023** ^b^
Mean (SD)	0.44 (0.51)	0.19 (0.44)	
Range	(0–1)	(0–2)	
Number of days at home			0.112^b^
Mean (SD)	16.39 (5.97)	18.74 (4.43)	
Range	[2–21]	[3–21]	

^a^ Fisher's exact.

^b^ Wicoxon rank‐sum.

Further analysis utilizing post hoc classification of readmissions as non‐preventable (*n*), possibly preventable (pp), or preventable (p) revealed that the the control group consisted of 6 non‐preventable readmissions (75%) and 2 possibly preventable readmissions (25%) while the intervention group consisted of 6 non‐preventable readmissions (54.5%), 4 possibly preventable readmissions (36.3%) and 1 preventable readmission (9%). In result, the proportion of possibly‐preventable or preventable readmissions in the intervention group (45.5%) was greater than the proportion in the control group (25%). However, there was a decrease in the number of non‐preventable readmissions in the intervention group (75% in the control versus 54.5% in the intervention group). These The reasons for re‐admission in each group and post hoc classification are listed in Table S3.

Caregiver/patient distress, as measured by the NCCN distress thermometer [21] with a scale of 0–10, on days 8, 15, 22 and 29 is shown in Table 5. No statistically significant difference was found between the control and intervention groups, although within each group there was a statistically significant decrease in distress over the 3‐week study period. In the control group, the mean distress decreased from 6.5 on day 8 to 4.33 on day 29 (*p* = 0.014), and similarly in the intervention group, the mean distress decreased from 5.46 on day 8 to 3.39 on day 29 (*p* = 0.002). Of the total responses, 200 (79%) were from a mother, 42 (17%) were from a father, 1 (0.4%) was from a grandmother, and 9 (4%) were from the patient themselves.

**TABLE 5:** Patient Results – NCCN Distress Thermometer.

	Control group (*N* = 64 responses)	Intervention group (*N* = 137 responses)	*p* (between groups)
Day 8			0.222^a^
*N*	16	35	
Mean (SD)	6.50 (2.78)	5.46 (3.16)	
Range	1–10	(0–10)	
Day 15			0.202^a^
*N*	16	33	
Mean (SD)	4.75 (3.42)	3.58 (2.36)	
Range	1–10	(0–9)	
Day 22			0.373^a^
*N*	17	38	
Mean (SD)	4.47 (3.22)	3.68 (2.83)	
Range	(0–10)	(0–9)	
Day 29			0.282^a^
*N*	15	31	
Mean (SD)	4.33 (3.04)	3.39 (2.53)	
Range	1–9	(0–9)	
*p* (within groups)	0.014^a^	0.002^a^	

^a^ GEE.

The median number of survey responses per patient was 80% ± 32% with a range of 0%–100%. Exploratory analysis was conducted to identify potential risk factors for lower rates of response to the surveys. Patients were dichotomized into two groups based on the median response rate for the entire sample: patients completed > 80% of surveys (High Responders) and those who completed < 80% of surveys (Low Responders). Low Responders were found to be less likely to identify as Hispanic ethnicity (*p* = 0.024), but no other demographic or treatment variables were significantly different between the groups, including Spanish vs. English speakers (Table 6). Low Responders' number of admissions was not significantly different from that of High Responders with a mean admission number of 0.14 ± 0.35 for Low Responders versus 0.24 ± 0.51 for High Responders (*p* = 0.469).

**TABLE 6:** Analysis of Low Responders versus High Responders.

	Low responders (*N* = 29)	High responders (*N* = 29)	*p*
Sex			0.791^a^
Male	17 (58.6%)	16 (55.2%)	
Female	12 (41.4%)	13 (44.8%)	
Race			0.670^b^
Caucasian	24 (82.8%)	27 (93.1%)	
African American	1 (3.4%)	0 (0%)	
Asian	1 (3.4%)	1 (3.4%)	
Native American	2 (6.9%)	0 (0%)	
Other/Unknown	1 (3.4%)	1 (3.4%)	
Ethnicity			**0.024** ^b^
Hispanic	5 (17.2%)	14 (48.3%)	
Non‐Hispanic	24 (82.8%)	15 (51.7%)	
Preferred Language			0.103^a^
English	26 (89.7%)	20 (69%)	
Spanish	3 (10.3%)	9 (31%)	
Type of ALL			0.423^b^
B cell	24 (82.8%)	27 (93.1%)	
T cell	5 (17.2%)	2 (6.9%)	
Treatment Protocol			0.561^b^
AALL1731	15 (51.7%)	14 (48.3%)	
AALL1732	10 (34.5%)	13 (44.8%)	
AALL1231	4 (13.8%)	2 (6.9%)	
# of drugs in induction			0.793^a^
3‐drug induction (AALL 1731)	15 (51.7%)	14 (48.3%)	
4‐drug induction (AALL 1732 or AALL 1231)	14 (48.3%)	15 (51.7%)	
Age at diagnosis			0.913^c^
Mean (SD)	8.35 (5.54)	8.63 (6.79)	
Number of Admissions			0.469^c^
Mean (SD)	0.14 (0.35)	0.24 (0.51)	
Days Readmitted			0.539^c^
Mean (SD)	1.31 (3.84)	1.76 (4.47)	

^a^ Chi‐square.

^b^ Fisher's exact.

^c^ Wicoxon rank‐sum.

On average, there were only a few symptoms reported per patient per day, and our team performed a total of 1–3 calls per day based on the flagged responses of participants in the survey. This number of calls per day was not significantly burdensome to the medical team as determined through verbal feedback on a weekly basis, thereby demonstrating the sustainability of this daily symptom home monitoring system. The survey interface provided a free text box for families to express specific concerns not addressed in the questions (see Table 7 for selected examples). This free‐text option allowed for direct communication between families and the medical team, thereby addressing concerns not specifically addressed via the survey questions. For example, a patient reported not having stooled in over 48 h over the weekend, prompting a call from the team and was given instructions on how to increase their stooling regimen, thus potentially preventing a worsening problem that would have needed to be addressed at the clinic visit. Another family replied “no” when asked about compliance with chemotherapy medication, prompting a phone call. In this instance, the patient was suffering from intractable vomiting while taking medications. That patient was able to have a same day nursing appointment made for nasogastric tube placement, thus ensuring further compliance with chemotherapy medications.

**TABLE 7:** Examples of Free‐texted Responses from Families to the Medical Team.

Comment	Potential outcome
“This is not urgent and we're not even sure if you can help. Her hair is starting to fall out. Have you heard of ways that parents explain this to their young kids? She's so sad about it. We're just trying to find any way to make her understand or feel better about herself.”	Something the family was worried about that we might not have thought to address
“Her poop is getting more loose more like a diarrhea should I stop the constipation meds? She also got a butt sore last night.”	If not addressed could have led to an admission for dehydration
“Wanting to confirm the day we stop the Dex this weekend.”	Ensured the chemotherapy is correctly administered at home
“He has a dentist appt today. Is it still okay for him to go?”	Important for us to know in Induction therapy in order to guide the family that patient shouldn't have major dental work completed during Induction due to high infection risk
“He's having a lot of throat pain, to where he says he can't eat, but he is hungry.”	Could have helped to prevent an admission from malnutrition
“She wanted to tell you she is feeling happy and a lot better today.”	Improved family and medical team connection

Two patients (2.6%) passed away during the study, both in the intervention group. The first patient presented to clinic on day 15 of induction therapy with abnormal vital signs (fever, tachycardia, and tachypnea), increased work of breathing, and abdominal tenderness on exam. They were found to have septic shock secondary to *Bacillus Cereus* bacteremia and ultimately passed away despite maximal interventions in the PICU. In total, this patient had 5 days at home (and on study) after being discharged from their initial admission. The second patient presented to the emergency department on day 11 of induction therapy after experiencing a syncopal episode at home with loss of consciousness and resultant fall. During this patient's readmission, they were found to have a disseminated fungal infection with Rhizopus microsporus from which they ultimately passed away. In total, this patient spent 3 days at home (and on study) prior to their readmission.

## 4 Discussion

Despite high overall cure rates for childhood ALL, morbidity remains high, especially in the first month of treatment. Initial therapy includes a steroid backbone as well as multiagent chemotherapy which may cause multiple symptoms and complications, leading to increased re‐admission rates, more time spent in the hospital, worsening caregiver distress and a higher overall burden on patients and their family's wellbeing. We evaluated the use of a daily symptom home monitoring system for patients undergoing initial chemotherapy for Acute Lymphoblastic Leukemia utilizing a HIPAA‐compliant texting interface on the family's own cellular phones. We showed a significant decrease in readmissions among the intervention participants as compared to controls. Although intervention participants spent more days at home than the control group, the difference did not reach statistical significance. Of note, a higher proportion of patients received a 4‐drug induction versus a 3‐drug induction regimen in the control group compared to the intervention group, and patients receiving a 4‐drug induction may have been at higher risk for readmission due to their more intense therapy. Although this difference did not reach statistical significance in this study, the difference in baseline risk for readmission may have confounded our findings.

Both control and intervention groups completed a weekly questionnaire regarding caregiver distress. In contrast to our initial hypothesis that daily interactions between families and the study team may decrease caregiver distress, the two groups did not have significantly different reported distress. However, both groups reported a very high level of initial distress, highlighting the complexity and intensity of ALL induction therapy. In addition, both groups demonstrated a decrease in distress levels over the study period, with the mean distress level improved from 6.5 to 4.33 for the control group and from 5.46 to 3.39 for the intervention group (Table 5). These results were consistent with Peterson et al.'s study in 2020 which demonstrated caregivers of pediatric patients with newly diagnosed leukemias had an initial high mean level of distress (6.30 ± 2.59), which subsequently decreased 6 months later but remained above the clinical cut off score of 4 (4.41 ± 2.66) [24]. Although the intervention did not improve distress, it also did not appear to worsen distress and thus does not appear to be an additional burden for patients and/or their families. This is an important finding as our patients and their families are already adjusting to a new diagnosis with life‐altering implications and an intensive therapy regimen during the first month of treatment.

Another important and clinically relevant finding was regarding the level of burden this intervention placed on the medical team. Through weekly meetings during the study period to obtain verbal feedback, the dedicated nursing team shared that these calls were well received and helped form stronger therapeutic relationships with patients and their families that ultimately appeared to allow for smoother care during upcoming clinic visits. In addition, the nursing team shared that it allowed them to develop their problem‐solving skills and immensely added to their work satisfaction. Future studies that include more comprehensive measures to assess burden on the medical team are needed to explore the full impact of this intervention.

Compliance with the daily symptom survey was relatively high, with an 80% ± 32% response rate, and readmission rates did not significantly vary between high and low survey responders. This study was not designed to determine predictors of differential response rates, but families may have utilized the home monitoring interface more often with specific concerns (i.e., patient is not doing well and experiencing more symptoms) and not responding when the patient feels well and they have no concerns. This potential pattern of response may explain why low responders did not have higher readmission rates as those patients were doing well at home without any acute concerns.

The daily symptom home monitoring system was successfully deployed in Spanish as well as English. Hispanic ethnicity predicted high response rates to the daily texted survey (Table 6) indicating that patients in this ethnic group felt comfortable interacting with their health care team using this modality. One of the Spanish‐speaking mothers of a patient in the intervention group shared with our study team that she found the texted survey interface very accessible as it allowed for easy toggling between Spanish and English. In addition, she shared that utilizing the Spanish interface of the survey was less time consuming to communicate any acute concerns as she did not have to call and wait for an interpreter over the phone. This high response rate in our study to the daily texted survey for patients and their families of Hispanic ethnicity is intriguing as current literature on determinants of health in patients with ALL overall reports decreased healthcare engagement with decreased adherence to medication and significantly inferior outcomes in Hispanic patients [25, 26]. We believe that the ability to easily toggle to Spanish is a major strength of our daily symptom home monitoring system as many studies do not encompass non‐English speakers and our study not only included the Spanish speaking population but also appeared to improve communication.

The intervention was not designed to prevent deaths, and in fact 2 patients (2.6%) died during the study period. This is within previously reported ranges [27, 28, 29, 30]. Other interventions to decrease mortality need to be developed.

Limitations of the study include a small sample size from a single institution. A larger multi‐institutional study to enroll more patients and to increase the statistical power of the study is needed. Additionally, a per‐patient randomization would be the optimal study design to reduce any potential bias and balance participant characteristics between groups, but it was felt to lead to too much confusion and potential bias within the health care team. Lastly, participants were required to own a smartphone to enroll in this study, and while ubiquitous in modern society, this requirement may result in inequities by social determinants of health.

Over recent years, there has been an increase in the application of technology and artificial intelligence in the medical field. This daily symptom home monitoring texted survey is an example of a home monitoring system that is flexible and could be tailored to other treatment phases or disease states as a cost‐effective and simple intervention to improve patient outcomes. Other ALL treatment phases in particular that may benefit from this intervention are intensified consolidation, intensified delayed intensification (DI), and patients receiving a four‐drug induction with dexamethasone as these treatment phases have been shown to have a significantly higher number of hospitalizations (76.2% of patients receiving intensified consolidation hospitalized vs. 32.3% receiving standard consolidation, 72.9% of patients receiving intensified DI hospitalized vs. 50.3% receiving standard DI, and patients receiving four‐drug induction with dexamethasone had an 85% higher rate of ED visits and 44% higher rate of hospitalization compared to those receiving prednisone) [22]. This intervention may also prove useful to other non‐oncological disease states, such as the population of both pediatric and adult patients with sickle cell disease who have high emergency department utilization and hospitalization rates [31, 32]. With this intervention, improved patient outcomes may include decreased hospitalizations and length of hospital stays, lower costs for both patients and the healthcare system, and improved emotional and logistical burden for patients and their families that are associated with disease and/or therapy related complications. Although our study did not specifically measure or show decreased symptom burden as an improved patient outcome, future studies may reveal this if measured. Additionally, outside of a clinical trial setting, these services could be billed under patient home monitoring codes [33].

In conclusion, our results of the use of a simple, HIPAA‐compliant texting interface to monitor daily symptoms at home indicate a significantly reduced readmission rate in newly diagnosed ALL patients undergoing induction therapy. Analysis of this home monitoring system on participant and/or caregiver's satisfaction and connection to the medical team is currently ongoing. Future multi‐institutional trials—enrolling more patients, including other ALL treatment phases and in targeted populations with a higher risk for admission rates, and focusing on evaluation of health care cost savings as well as predictors of response rates—need to be conducted.

## Author Contributions

**Rotem Fishel Ben‐Kenan:** data curation (equal), formal analysis (equal), investigation (lead), methodology (equal), project administration (equal), visualization (equal), writing – original draft (lead), writing – review and editing (lead). **Laura L. Retson:** conceptualization (equal), data curation (equal), methodology (equal), supervision (equal), writing – review and editing (equal). **Briana Fodor:** data curation (equal), funding acquisition (equal), methodology (equal), project administration (equal). **M'hamed Temkit:** formal analysis (lead), software (equal), visualization (equal), writing – review and editing (supporting). **Alexandra Walsh:** conceptualization (lead), data curation (equal), formal analysis (equal), funding acquisition (lead), investigation (equal), methodology (equal), project administration (equal), supervision (lead), writing – review and editing (lead).

## Funding

Leadership Circle, Phoenix Children's Hospital Foundation and the generous continued support of the PCH Foundation through direct funding to Center for Cancer and Blood Disorders at Phoenix Children's Hospital.

## Conflicts of Interest

The authors declare no conflicts of interest.

## Supporting information


**Appendix S1:** Full Text of Administered Surveys.

**Appendix S2:** Patient Outcomes in 3‐drug versus 4‐drug Induction.
**Appendix S3:** Analysis of Re‐admissions in the Control and Intervention Groups.

## Data Availability

The data that support the findings of this study are available on request from the corresponding author. Restrictions apply to the availability of these data, which were used under license for this study.
