# Supplementary material for: Daily Symptom Home Monitoring Decreases Hospital Readmissions in Children and Young Adults With Acute Lymphoblastic Leukemia
Source: Cancer Med. 2026 Apr 8;15(4):e71719. doi: 10.1002/cam4.71719 (PMC13061743; doi:10.1002/cam4.71719)

Supplement 1. Full text of administered surveys

Daily Questionnaire:


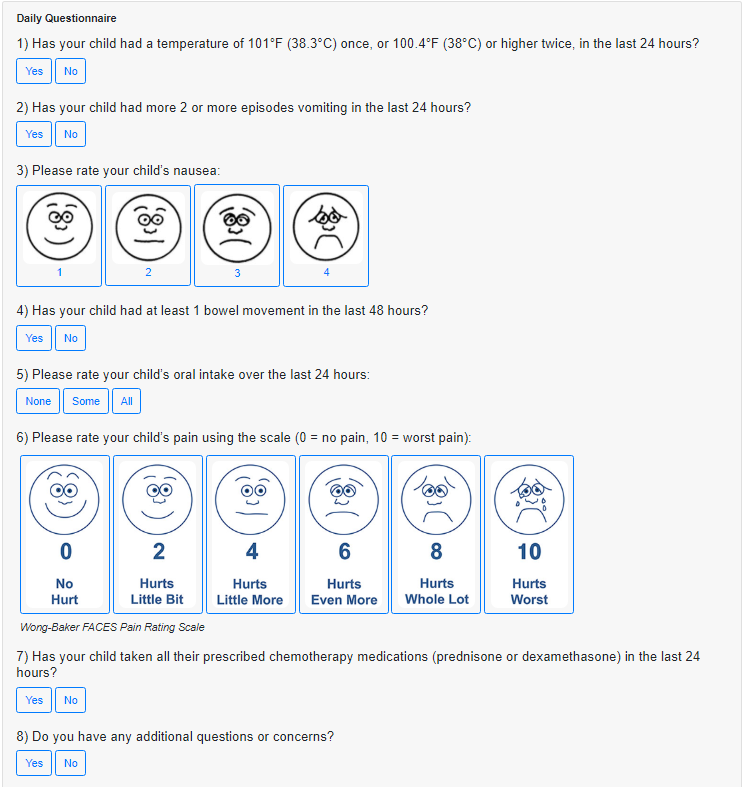


Triggers for immediate action based on answers from Daily Questionnaire noted above:


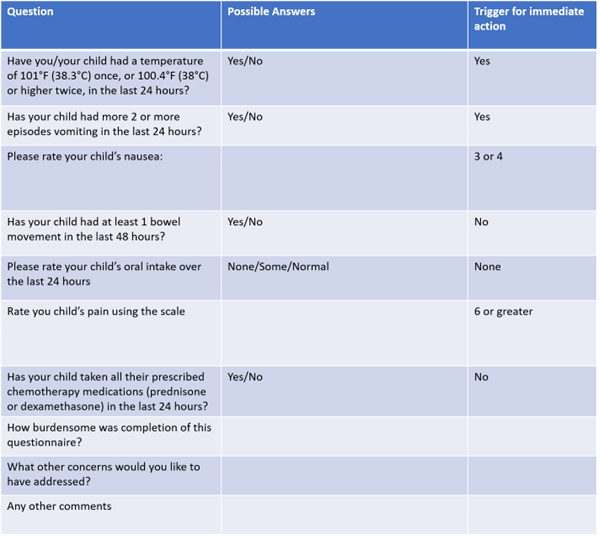


Weekly questionnaires regarding caregiver distress:

- Included the Client Satisfaction Questionnaire (CSQ), TBI-Care QOL Caregiver Stress and NCCN Distress Thermometer Question


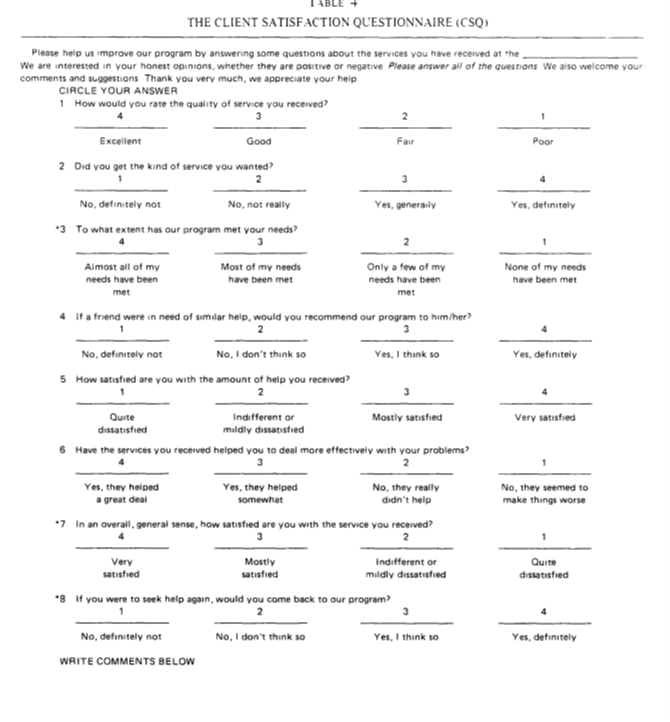


TBI-Care QOL Caregiver Stress


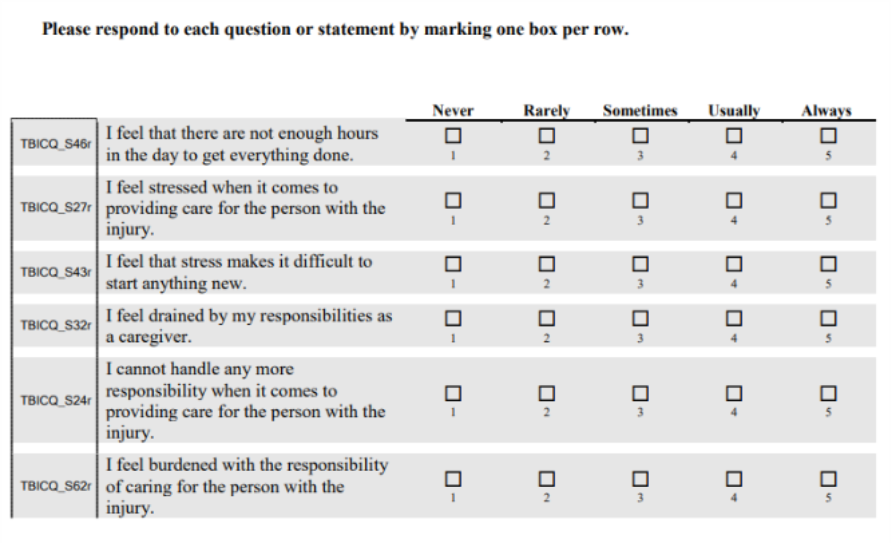


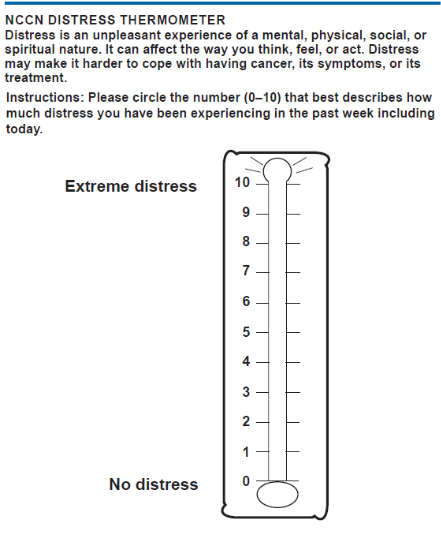

Supplement: Supplementary file 1 — Appendix S1: Full Text of Administered Surveys. [file CAM4-15-e71719-s001.docx]
