# Supplementary material for: Daily Symptom Home Monitoring Decreases Hospital Readmissions in Children and Young Adults With Acute Lymphoblastic Leukemia
Source: Cancer Med. 2026 Apr 8;15(4):e71719. doi: 10.1002/cam4.71719 (PMC13061743; doi:10.1002/cam4.71719)
Supplement: Supplementary file 2 — Appendix S2: Patient Outcomes in 3‐drug versus 4‐drug Induction. Appendix S3: Analysis of Re‐admissions in the Control and Intervention Groups. [file CAM4-15-e71719-s002.docx]

Supplement 2. Patient Outcomes in 3-drug versus 4-drug Induction

| **Supplementary Table 2. Patient Outcomes in 3-drug versus 4-drug Induction** | | | |
| --- | --- | --- | --- |
|  | **3-drug Induction (AALL 1731)**  (N=36) | **4-drug Induction (AALL 1732 or AALL 1231)**  (N=40) | **P-value** |
| **Number of admissions per patient** |  |  | **0.0083^1^** |
| 0 | 32 (88.9%) | 26 (65%) |  |
| 1 | 3 (8.3%) | 14 (35%) |  |
| 2 | 1 (2.8%) | 0 (0%) |  |
| **Total admission number** |  |  | **0.0204^2^** |
| Mean (SD) | 0.14 (0.42) | 0.35 (0.48) |  |
| Range | (0-2) | (0-1) |  |
| **Number of days at home** |  |  | **0.0037^2^** |
| Mean (SD) | 19.36 (3.90) | 17.13 (5.48) |  |
| Range | (2-21) | (3-21) |  |
| ^1^ Fisher's exact ^2^ Wicoxon rank-sum | | | |

Supplement 3. Analysis of Re-admissions in the Control and Intervention Groups

| Supplementary Table 3a. Analysis of Re-admissions in Control Group | | |
| --- | --- | --- |
| **Control group re-admission event** | **Primary reason for re-admission** | **Non-preventable (n), possibly preventable (pp) or preventable (p)** |
| 1 | Fever | n |
| 2 | Diarrhea | pp |
| 3 | Fever | n |
| 4 | Fever | n |
| 5 | Loose stools | pp |
| 6 | Seizures | n |
| 7 | Fever | n |
| 8 | Fever | n |
|  |  |  |
| Supplementary Table 3b. Analysis of Re-admissions in Intervention Group | | |
| **Intervention group re-admission event** | **Primary reason for re-admission** | **Non-preventable (n), possibly preventable (pp) or preventable (p)** |
| 1 | Pneumothorax & pneumomediastinum | n |
| 2 | Hyperglycemia | p |
| 3 | Fever | n |
| 4 | Abdominal pain | pp |
| 5 | Tachypnea | n |
| 6 | Fever | n |
| 7 | Abdominal pain & decreased oral intake | pp |
| 8 | Fainting episode | pp |
| 9 | Fever | n |
| 10 | Tachypnea | n |
| 11 | Abdominal pain & vomiting | pp |
